# β-adrenergic receptor responsiveness in aging heart and clinical implications

**DOI:** 10.3389/fphys.2013.00396

**Published:** 2014-01-09

**Authors:** Nicola Ferrara, Klara Komici, Graziamaria Corbi, Gennaro Pagano, Giuseppe Furgi, Carlo Rengo, Grazia D. Femminella, Dario Leosco, Domenico Bonaduce

**Affiliations:** ^1^Department of Translational Medical Sciences, University of Naples “Federico II”Naples, Italy; ^2^“S. Maugeri” Foundation, Scientific Institute of Telese Terme (BN), IRCCSTelese Terme, Italy; ^3^Department of Medicine and Health Sciences, University of MoliseCampobasso, Italy

**Keywords:** β-adrenergic receptors, β-adrenoceptor desensitization, β-adrenoceptor down-regulation, G-protein coupled receptor kinase, aging heart, failing heart, exercise

## Abstract

Elderly healthy individuals have a reduced exercise tolerance and a decreased left ventricle inotropic reserve related to increased vascular afterload, arterial-ventricular load mismatching, physical deconditioning and impaired autonomic regulation (the so called “β-adrenergic desensitization”). Adrenergic responsiveness is altered with aging and the age-related changes are limited to the β-adrenergic receptor density reduction and to the β-adrenoceptor-G-protein(s)-adenylyl cyclase system abnormalities, while the type and level of abnormalities change with species and tissues. Epidemiological studies have shown an high incidence and prevalence of heart failure in the elderly and a great body of evidence correlate the changes of β-adrenergic system with heart failure pathogenesis. In particular it is well known that: (a) levels of cathecolamines are directly correlated with mortality and functional status in heart failure, (b) β_1_-adrenergic receptor subtype is down-regulated in heart failure, (c) heart failure-dependent cardiac adrenergic responsiveness reduction is related to changes in G proteins activity. In this review we focus on the cardiovascular β-adrenergic changes involvement in the aging process and on similarities and differences between aging heart and heart failure.

## Introduction

Epidemiological studies reveal an high incidence and prevalence of heart failure in the elderly (Roger et al., 2011). In chronic heart failure substantial and characteristic changes occur in the cardiac structure and function and these modifications are not very different from those observed in the aging heart (Shioi and Inuzuka, 2012). The peculiar age-related cardiac structural changes are represented by an increase in cardiomyocyte size and in myocardial thickness (Scholtz et al., 1988; Olivetti et al., 1991), which are able to affect the contractile efficiency of the left ventricle. These changes are associated with increased cardiac fibrosis and vascular stiffening. However, epidemiological and autopsy-based studies, performed in subjects free from coronary artery disease and hypertension, have demonstrated no significant age-related changes in cardiac mass in elderly female and a decrease in left ventricular mass in elderly male compared to young male (Hess et al., 2002; Khouri et al., 2005) (“cardiac sarcopenia”). Nevertheless, ageing is not associated with impaired systolic cardiac function at rest, as demonstrated by echocardiographic and radionuclide studies performed in normotensive healthy subjects (Khouri et al., 2005). Differently, ageing is related to diastolic left ventricle function with increased prevalence of diastolic heart failure. It is well known that in healthy elderly there is a reduction in left ventricle inotropic reserve and exercise tolerance. Reduced inotropic cardiac reserve is thought to be related to increased vascular afterload, arterial-ventricular load mismatching, physical deconditioning and impaired autonomic regulation (so called “β-adrenergic desensitization”). It is interesting to point out the similarity observed in terms of hemodynamic profile under adrenergic challenge between younger β-blocked subjects and healthy elderly subjects without β-blocker treatment (Fleg et al., 1994). Adrenergic receptors activation by catecholamines is the most important regulatory mechanism of cardiovascular performance. Adrenergic receptor agonists, as well as exercise, stimulate the adrenergic system increasing heart rate, myocardial contractility and relaxation, reducing left ventricular afterload and redistributing blood flow to skeletal muscle. Anyway, adrenergic responsiveness is altered with aging (White et al., 1994). In fact, both animal and human studies indicate a decline in heart rate, cardiac contractility, cardiac output and ejection fraction in response to β-adrenergic stimulation and exercise (Rinaldi et al., 2006; Corbi et al., 2012a,b). Part of the age-related decline in β-adrenergic responsiveness has been attributed to a general decrease in cardiac contractility. However, several observations indicate a crucial role of reduced β-adrenergic receptor density and some defects involving the adenylyl cyclase cascade beyond β-receptor levels (Ferrara et al., 1995, 2005; Freedman et al., 1995). The age-associated reduction in maximal heart rate during high levels of exercise are in relationship with a reduced β-adrenergic responsivity despite an increase in circulating levels of catecholamines (Corbi et al., 2013a). Aging is associated with elevated neuro-hormonal activation, and characterized by elevated plasma norepinephrine and epinephrine circulating levels, due to increased spillover from tissues (including the heart) and reduced plasma clearance of catecholamine (Ng et al., 1993; Esler et al., 1995). The “β-adrenergic desensitization,” at least in part, is due to the reduction of β-adrenergic receptor plasma membrane density described in hearts of both senescent animals and elderly humans (White et al., 1994; Xiao et al., 1998). The β-adrenergic receptors are members of the G-protein-coupled receptor family, which acts by coupling with guanine nucleotide binding proteins, and the age-induced decrease in β-adrenoceptor responsiveness is characterized at the molecular level by decreased activation of adenylyl cyclase and reduced production of cAMP. Beside β-adrenergic receptor down-regulation, another crucial age-related alteration of this signaling pathway seems to be the coupling of the β-adrenergic receptor to adenylyl cyclase via the G_*s*_ protein, which leads to a reduction in the ability to increase cAMP and to activate protein kinases. Some studies have also reported an increase in G_α*i*_ activity as a possible additional mechanism in “β-adrenergic desensitization.” Moreover, the reduction in the efficacy of cardiac β-adrenoceptor stimulation with aging could be also related to other mechanisms, such as the upregulation of G protein–coupled receptor kinases (Rengo et al., 2012a), whereas the role of these kinases in aging heart is controversial. From an overall data analysis on the role of aging in β-adrenoceptor regulation in human and animal hearts it is possible to conclude that the reduced response to β-agonists is common to all species and all cardiac tissues investigated. Moreover, the age-related changes are limited to β-adrenoceptor-G-protein (s)-adenylyl cyclase system abnormalities, while the type and level of abnormalities change with species and tissues. These differences could explain the inconsistency in results obtained in different experimental models of aged heart. Interestingly, several evidence suggest that the β-adrenergic receptor system plays an important role also in heart failure pathogenesis. In fact, it is well known that (a) the levels of cathecolamines are directly correlated with mortality and functional status in heart failure, (b) cardiac β-receptors, in particular β_1_ subtype, are downregulated in heart failure and (c) heart failure-dependent cardiac adrenergic responsiveness reduction is related to adrenoreceptor kinases and G_α*i*_ increased activities.

## Aims

This review focuses on (a) the development of knowledge on aging heart over the years, (b) the changes involving the sympathetic system in relationship to the cardiovascular aging in different species, (c) the clinical implications of changes in β-adrenergic mechanisms in the aging heart and d) the similarity between aging and failing heart.

## β-adrenergic signaling in the heart at molecular level

For the first time the existence of β-adrenergic receptors (β-AR) was described in 1948 by Alquist (1948). At the present three subtypes of β-AR: β_1_-AR, β_2_-AR, β_3_-AR have been recognized. A fourth subtype has been proposed and investigations have been recently clarified its functioning and localization (Lewis et al., 2004). At the beginning it was thought that only β_1_-AR subtype was expressed in the cardiac cells. However, many studies provided evidence that both heart β_1_-AR subtype and β_2_-AR subtype (Lemoine and Kaumann, 1991; Altschuld et al., 1995; Lonardo et al., 2005), coexist in humans as well as in animals. In the human heart approximately 80% of the β-AR subtype expressed belong to the β_1_-AR, followed by 20% of the β_2_-AR subtype (Lakatta and Levy, 2003). It is important to underline that this β-AR expression proportion has been observed in the non-failing young, but not in elderly human heart.

Modern molecular biology techniques and radio-ligand binding studies have shown that major expression and main contribution in the contractile functioning of cardiac cells belong to the β_1_-AR subtype (Benovic et al., 1991; Borea et al., 1992). In humans, as well as in other animals with relative big body weight like sheep, dogs or cats, the β_1_-AR and β_2_-AR are both significantly present, while in other small animals like rats or guinea-pigs the presence of β_2_-AR is undetectable. Some other studies found the presence of β_2_-AR in rat hearts but not localized in ventricular myocytes (Buxton and Bruton, 1985). Even the different specie-dependent contribution of β-AR and the difficulties to find a perfect experimental model, it is well known that in all different species the general mechanical pathway is always related to adenylyl cyclase (AC) activation, cyclic AMP (c-AMP) formation, Protein Kinase A (PKA) activation and G-Protein Coupled Receptor Kinase (GRK) activation. β-AR are members of the G-Protein Coupled Receptors (GPCRs) family which acts by coupling with Guanine nucleotide binding proteins (Rengo et al., 2012a). β_1_-AR subtype is coupled to the stimulatory G protein (G_*s*_). G_*s*_ protein is a heterotrimetric protein made up of α, β, and γ subunits. The presence of β-AR agonists induces the dissociation of G_*s*_ protein in two subunits: α subunit and β−γ subunit. The primary effect of this dissociation is the activation of AC that catalyzes the conversion of ATP to c-AMP, a second intracellular messenger, and induces the activation of c-AMP dependent PKA. Serine and threonine residues of many regulatory proteins are phosphorylated by PKA. These regulatory proteins include: β-AR themselves, myofilament proteins (troponine I and C protein), membrane proteins (phospholamban—PLB, L-type Ca^++^ channels, Sarcoplasmatic Reticulum—SR, Ca^++^/ATPase inhibitory protein). The stimulation of β-AR modifies not only the cardiac excitation and contraction but also other cellular functions such as gene transcription and growth, and can induce death. An important role for the above mentioned functions has played by the activation of Mitogenic-Activated Protein Kinase (MAPK). Moreover, these kinases are thought to be implicated in the regulation of several vital cellular processes, including differentiation, proliferation, growth, and death (Van Biesen et al., 1995). Ultra Violet light, osmotic stress and heat shock can activate MAPK signaling cascades and GPCRs play a pivotal role in the regulation of MAPKs, particularly of the extracellular signal-regulated kinase (ERK_1/2_) MAPK. One major pathway of GPCRs-mediated activation of MAPKs is dependent on “transactivation” of a group of receptor tyrosine kinases, such as epidermal growth factor and insulin-like growth factor. Additionally the activation of p38 MAPK, also called “stress-activated protein kinase,” is associated with the initial signs of cardiac hypertrophy in response to “*in vivo*” pressure overload or ischemic/reperfusion injury (Bogoyevitch et al., 1996; Wang et al., 2013).

The β-AR stimulation induced by catecholamines is also responsible for the Ca^++^ influx, that by itself triggers a potential release of Ca^++^ from SR, acting on the ryanodine receptors. The intracellular Ca^++^ release activates contractile proteins, finalizing the muscular contraction (positive inotropic effect). Then, intracellular Ca^++^ is removed from the cytoplasm by the SR-Ca^++^/ATPase pump and the Na^+^/Ca^++^ exchange. The further acceleration of Ca^++^ removing leads to the muscle relaxation. The maximum velocity of relaxation is defined as positive lusitropic effect. As a result of PKA activation, the β-AR stimulation triggers the G-Protein Coupled Receptor Kinase family, like GRK2. GRKs are a family of serine/threonine protein kinases that phosphorylates GPCRs only when the receptors are in the activated (agonist-bound) state. When β-ARs are stimulated by agonists, the β−γ subunits G interact with GRK_2_ bringing the kinase from the intracellular to the transmembranic localization, phosphorylating the β-ARs, becoming target for binding of β-Arrestin proteins. The β-Arrestin bounds to these receptors and prevents their further coupling to the G-protein, reducing the level of functional receptors, inducing the internalization of receptors and, as final result, their decreased density and desensitization (Freedman et al., 1995).

“*In vitro*” studies showed further mechanisms induced by stimulation of β_1_-AR. For example, persistent stimulation of β_1_-AR is able to activate Calmoduline Dipendent Kinase II, without the implication of PKA pathway. This mechanism induces cardiomyocyte hypertrophy and could explain the well-known relationship between adrenergic stimulation and cardiac hypertrophy^23–24^ (Ramirez et al., 1997; Morisco et al., 2000). In addition to the cardiac effects, β_1_-ARs regulate the release of renin, the activation of Renin-Angiotensine-Aldosteron (RAA)-system and the lipolysis.

β_2_-AR, one of the first receptors identified, belongs to the GPCRs family and plays an important role in the cardiovascular and respiratory physiology. Its main effects are related to vasodilatation and bronchodilatation (Corbi et al., 2013b). In addition β_2_-AR is responsible for glycogenolysis (Corbi et al., 2002) and relaxation of uterine muscle. Despite similarities β_1_-AR and β_2_-AR present different signaling pathways. The β_2_-AR is coupled to the G_*s*_ protein and to the G_*i*_ protein too (dual coupling of β_2_AR to G_*s*_ and G_*i*_ protein). There is also evidence that β_2_-AR signaling is coupled to an independent pathway like the Na^+^/H^+^ exchanged regulatory factor (Hall et al., 1998).

The effects of β_2_-AR G_*s*_ stimulation are not identical to them obtained from β_1_-AR stimulation. However, similarly to β_1_-AR, β_2_-AR-G_*s*_ stimulation increases the c-AMP and PKA activity. Recent studies have demonstrated that the effect of β_2_-AR c-AMP/PKA stimulation is limited to the subsurface membrane of the L-type Ca^++^ channels without cellular signal transmission. As a result of this mechanism it is observed a positive inotropic effect without influence on the intracellular Ca^++^ transient decay time, changes in myofilaments sensitivity to Ca^++^ and increase SR Ca^++^ uptake. Obviously β_2_-AR stimulation does not affect the relaxation time (lusitropic effect) as β_1_-AR does (Kuschel et al., 1999; Xiao et al., 1999).

Surprisingly the Ca^++^ influx, the PKA and the c-AMP levels apparently do not show significant association after β_2_-AR stimulation in studies using adult rat and canine models. Even if the reason of this dissociation remains unclear, the role of c-AMP modulation in the contractility of cardiac muscle is well established. In heart animal models forskolin induces c-AMP levels augmentation increasing the inotropic effect. Moreover, it has well demonstrated that the Ca^++^ influx mechanism is exclusively mediated by c-AMP pathway (Xiao et al., 1994).

On the other hand, the G_α*i*_ protein subunit inhibits the adenylcyclase enzyme activity. The β_2_-AR G_*i*_ signaling inhibits the c-AMP synthesis and has negative effects on the PKA activation. Persistent activation of β_2_-AR-G_β−γ*i*_ signaling activates in turn the phosphoinositol3- Kinase (PI3-K), an important downstream messenger that triggers the antiapoptotic factor Akt and seems to have a cardioprotective role. (Zhu et al., 2001; Cannavo et al., 2013).

The model of β_2_-AR dual coupling of to multiple G protein (G_*i*_ and G_*s*_ protein), is not well clarified. Several evidence indicate that β_2_-AR–G_*i*_ signaling compartmentalizes the β_2_-AR-G_*s*_–c-AMP signaling. Disrupting the G_*i*_ functioning by a potent G_*i*_ inhibitor like Pertussis Toxine (PTX) induces an enhance in the phosphorylation of PLB and an increased inotropic effect after β_2_-AR stimulation. In this occasion the β_2_-AR signaling is comparative to the β_1_-AR signaling^31^ (Xiao et al., 1995).

The β_2_-AR phosphorylation by PKA and GRK_2_ switches the β_2_-AR receptor coupling from G_*s*_ to G_*i*_. As demonstrated in several studies, the β_2_-AR-G_*i*_ coupling is suppressed after GRK_2_ activity inhibition. In a near future, it may be possible to prevent important structural changes, like myocardial stiffness, reactive fibrosis and remodeling present in the aging and failing heart, modifying the GRK_2_ and the G_*i*_ activated or inhibited status.

Another candidate mechanism, underling the compartmentalization of β_2_-AR–G_*s*_-c-AMP-PKA signaling in response to the β_2_-AR-Gi coupling, is the structural restriction of PKA diffusion by muscle specific protein A kinase anchory proteins (AKAP) (Enns et al., 2009). The phosphorylation of AKAP plays multiple roles including: ions influx, contraction, transcription of different genes, phosphorylation of multiple intracellular targets in cardiac myocytes including the L-type Ca^++^ channel in the sarcolemma, the ryanodine receptor (RyR_2_), and phospholamban in the SR. Deficiencies in this pathway have been linked to cardiomyopathy in humans, due to reduced phosphorylation of downstream targets such as cardiac troponin (McConnachie et al., 2006). Moreover, in genetically manipulated models it is obtained an increased positive inotropic effect after disrupting the APAK kinase anchory protein (Marshall, 1995; Spindler et al., 2013).

## Age-induced changes in the β-AR signaling

Adrenergic signaling is a very important for cardiovascular physiology. In conditions involving physical or psychological stress high levels of cathecolamines like norepinephrine and epinephrine are released from the adrenal medulla. It is well known that the action of cathecolamines is mediated by adrenergic receptors and the effects on cardiovascular system include: increased heart rate and myocardial contractility force and relaxation, increased cardiac output, reduced left ventricular afterload, a diversion of blood flow from the skin and splanchnic vessels to those supplying skeletal muscles, bronchial dilatation and a decline in metabolic activity (Young and Landsberg, 1998). Generally the age-related decrease in β-adrenoceptor response has been explained by a mechanism called “β-adrenoceptor desensitization.” It is a process characterized by β-AR molecular changes: phosphorylation of receptor structures enhanced by an agonist-receptor bind state, that induces the reduction of receptors density and their internalization. This process is well-described also in the heart failure (Rengo et al., 2012a). By aging a post-synaptic “β-adrenoceptor desensitization” responsible for a reduction in the autonomic modulation of the cardiac system, especially during physical exercise (Ehsani, 1987; Scarpace et al., 1994), is observed. During exercise the stroke volume increase is similar in young and older, but the mechanism of this increase is different. The elderly tends to augment stroke volume more through cardiac dilatation with an end-diastolic volume increase, while the young shows an increase in the ejection fraction without cardiac dilatation. Moreover, during exercise the older has a lower increase in heart rate and a greater raise in blood pressure (Ferrara et al., 2006; Corbi et al., 2012b). In particular in the aging human heart the maintaining of cardiac output during exercise is supported more heavily by the Franck-Starling mechanism and less by sympathetic stimulation demonstrating the presence of an age-related change in the adrenergic modulation. It is interesting to point out the similarity observed in terms of hemodynamic profile between younger β-blocked subjects and healthy elderly without β-blockers treatment in human models without HF (Fleg et al., 1994). Younger β-blocked subjects showed during exercise reduced heart rate and contractility index, and increased left ventricular volume, in short terms they apparently “looked older” but the results of β-AR blockade were greater in the young subjects compared to older ones, suggesting that β-AR responsiveness reduces with aging.

Interestingly, Leosco et al. (2007) demonstrated that exercise training alone as well as metoprolol alone or in combined therapy (exercise + metoprolol) improved the β-AR signaling in the aged heart suggesting a similar effect on β-AR signaling of chronic treatment with β-blockers and chronic exercise training. Furthermore they found an increased β-AR density inducing a reversible level of β-AR desensitization. The overall reduction in cardiac reserve is responsible for the decreased exercise tolerance and for the impairment in cardiac response at exercise in terms of reduced ejection fraction at peak and heart rate responsiveness during dynamic exercise. The responsibility of this response could be related to increased vascular afterload, arterial-ventricular load mismatching, reduced intrinsic myocardial contractility, physical deconditioning and impaired autonomic regulation (so called “β-adrenergic desensitization”). The mechanism of this impaired autonomic regulation induced by age is not completely clarified, although it is hypothesized that the increase in cathecolamines levels plays an important role in the “β-adrenergic desensitization” in aging and failing heart. In both conditions, as a result of a reduced plasma clearance and an increased spillover from the tissues, the level of circulating cathecolamines further rises. This prolonged cathecolamines action seems to be also related to the age-dependent reduction of the cathecolamines re-uptake transporter localized in the sympathetic nerve terminals (Leineweber et al., 2002). However, controversial opinions exist in the present literature about the changes in the systemic norepinephrine levels with age and the relationship between the age-related increased levels of cathecolamines and the suggested mechanisms of age-related “β-adrenergic desensitization” (Folkow and Svanborg, 1993; Esler et al., 1995).

The age-related “β-adrenergic desensitization,” a possible adaptive mechanism, has been observed in both animals and humans. An age-related effect on the maximum contractility response to isoproterenol has been demonstrated in arterially perfused interventricular septa from adult and senescent rats (Froehlich et al., 1978), and a reduced inotropic response of aged myocardium to catecholamines has been found in superfused trabeculae (Jiang et al., 1993). At molecular level, in particular, the decrease in β-adrenoceptor responsiveness has been related to changes in G-proteins and kinases activity even if differences in the level and extent of these changes exist among different species. Concerning the β-AR density, the first studies performed in circulating lymphocytes did not show any important age-related changes in β-AR density (Abrass and Scapace, 1981; Landmann et al., 1981). Also other data obtained from young and old rats did not report any changes in β-AR number (Bohm et al., 1993) did not find any changes in β-AR number but noticed a G_*i*_ increased content. This study hypothesized that G_*i*α_ might serve as a age-related regulator of cardiac AC activity in the absence of β-adrenoceptor changes. Interestingly Gudmundsdottir et al. (1991) showed that dietary fat and age modified the density of Ca^++^ channels and reduced the β-AR number in the rats.

Cerbai et al. (1995) confirmed that β_2_-AR as well as β_1_-AR are both functionally present in rat hearts, but only the β_1_-AR density was reduced with aging. In human aging heart White et al. (1994) found that the β_1_-AR down regulation mechanism was linked to the reduced number of β_1_-AR in a high affinity agonist binding state. In our experience, using a model of myocytes obtained from human failing (donors) and non-failing hearts (small biopsies from elderly and young patients undergoing coronary vein graft with preserve ventricular function), the contractile response to β-adrenoceptor stimulation has been found to be strongly reduced in single myocytes from failing human ventricle, but a part of this reduction was statistically related to the patient's age (Dobson et al., 1990; Davies et al., 1996). At beginning, it was thought that the desensitization mechanism of β-AR in the elderly is related to the increased levels of adenosine because its clear antiadrenergic action in the heart, as suggested by studies performed in guinea pig aging hearts. However, utilizing single isolated left ventricular cardiac myocytes from hearts of animals at different age, other studies showed that the age-related contractility impairment during β-adrenergic stimulation was confined to the β-adrenergic pathway with reduced net production of c-AMP, that could not be explained by the increased adenosine stimulation (Ferrara et al., 1995).

Ferrara et al. (1995), in a study performed on myocytes isolated from the young and old guinea-pigs, observed that there was a pronounced age-related decrease in contractility of myocytes after both isoproterenol and high Ca^++^ concentration stimulation. However, the Ca^++^ influence was less significant concluding that a general diminished contractility of single myocytes occurs during the physiological aging and these effects were more marked for β-AR stimulation than for high Ca^++^, suggesting a specific lesion in the AC related pathway. In a further investigation, using the same experimental model, the same authors (Ferrara et al., 1997a) studied the role of the inhibitory G-proteins (G_*i*_) in the decline of contractility related to β-stimulated activity. In these experiments they found that β-AR number was decreased by 27% in senescent animals and the G_*i*α_ activity, detected by PTX-catalyzed ADP ribosylation, was significantly increased in aged animals, while immunodetectable level of G_*i*α_ was not modified. The authors concluded that increased G_*i*α_ activity contributes significantly to the decreased response to β-AR stimulation in myocytes in aged guinea-pigs.

In different species (rats) Xiao et al. (1998) found a clear age-related reduction in contractility in response to β-AR subtype stimulation associated to a non-selective decrease in the density of both β-AR subtypes and a reduction in membrane AC response to either β_1_-AR or β_2_-AR stimulation. Moreover, the activity of β-AR kinase GRK_5_, and of G_*i*_ did not significantly change with aging, suggesting that the marked decreased response to β-ARs stimulation with aging in rat ventricular myocytes was linked to a decrease in both β-AR subtypes densities and a reduction in membrane AC activity, while neither GRKs nor G_*i*_ proteins appear to play a role in this mechanism. This apparent contrast with the results from Ferrara et al. (1997a) could be referred to the different animal models used for the experiments.

As in the failing heart, the role of chronic sympathetic drive in the β-AR desensitization in the aging heart can be hypothesized. Because the role of GRKs in the aging heart could be defined controversial same studies have been planned to focus on the role of GRKs in the aged cardiovascular system.

Schutzer et al. (2001) examined the correlation of GRKs level to age-related modifications in aorta of old rats. In particular they studied the age-related changes in distribution of GRK subtypes 2, 3, or 5 and β-arrestin (cytoplasmic vs. crude membrane preparations), and demonstrated that GRKs are implicated in the reduction of β-AR-mediated vasorelaxation with advancing age, suggesting a strong evidence that increased GRK activity plays an important role in cardiovascular physiology of aging. However, it is important to underline that in this study was not performed detection of GRKs in the heart tissues as it was performed in the previous studies mentioned above, explaining this controversial results. On this basis Leosco et al. (2003), studying the effects of exercise, found a reduced β-AR expression in aged rat carotid arteries, and the exercise itself restored the age-associated blunted β-AR responsiveness. This could explain with the age-related reduced adaptation of cardiovascular system to different stressors.

For the first time Leinweber et al. (2003) studied the possible GRKs activity alterations related to aging in humans. The study evaluated the cytosolic and membranous levels of GRKs in right atria from children with congenital heart disease undergoing cardiac surgery, elderly with coronary heart disease but not suffering from heart failure undergoing coronary artery by-pass grafting, and from a small group of elderly patients with heart failure also undergoing cardiac surgery. The main result was that neither cytosolic nor membranous GRKs activity were modified in elderly compared to children, while, as confirmed in previous studies (Ungerer et al., 1993; Ping et al., 1997; Vinge et al., 2001; Rockman et al., 2002; Iaccarino et al., 2005) there was a notable up-regulation in the GRKs activity in the failing heart. The reason because the β-AR desensitization in the aging heart is not associated to GRKs up-regulation is not well clarified at present. It can be hypothesized that the GRKs levels are more affected by acute triggers as in heart failure happens and not influenced by a gradual chronic “aging” process of β-adrenergic drive, or at least during this life-long process GRKs levels are associated to other adaptive mechanism that may influence their up-regulation. In Table 1 the main general age-related changes in β-adrenergic signaling are described.

**Table 1:** **Cardiovascular system: the main general age-related changes in β-adrenergic signaling**.

**Author**	**Constituent**	**Species**	**Changes**
Abrass and Scapace, 1981	β-AR density	Human (circulating lymphocytes)	Unchanged
Landmann et al., 1981	β-AR density	Human (circulating lymphocytes)	Unchanged
Gudmundsdottir et al., 1991	β-AR density	Rat (heart)	Decreased
Bohm et al., 1993	β-AR density	Rat (heart)	Unchanged
Bohm et al., 1993	G_*i*_ content	Rat (heart)	Increased
White et al., 1994	β_1_-AR density	Human (heart)	Decreased
Bazan et al., 1994	β-AR density	Rat (heart)	Unchanged
Cerbai et al., 1995	β-AR density	Rat (heart)	Decreased
Ferrara et al., 1995	β-AR density	Guinea-pig (heart)	Decreased
Ferrara et al., 1997a,b	G_*i*_ content	Guinea-pig (heart)	Unchanged
Ferrara et al., 1997a,b	G_*i*_ activity	Giunea-pig (heart)	Increased
Xiao et al., 1998	β_1, 2_-AR density	Rat (heart)	Decreased
Xiao et al., 1998	G_*i*_ activity	Rat (heart)	Unchanged
Schutzer et al., 2001	β-AR	Rat (aorta)	Decreased
Schutzer et al., 2001	GRK_2_, GRK_3_, β-arrestin	Rat (aorta)	Increased
Leinweber et al., 2003	GRK	Human (heart)	Unchanged

## Clinical implications of alterations in β-adrenoceptor mechanisms in the aging heart. relationship with heart failure

Heart failure and associated clinical implication induced by alterations in β-adrenoceptor mechanisms are a central problem for elderly population (Ferrara et al., 1997b). Epidemiological studies showed high prevalence and incidence of heart failure in the elderly (Corbi et al., 2008; Roger et al., 2011) due also to an increased longevity. The incidence doubles with each decade of life and prevalence rises almost 10% of those older than 80 years (Cacciatore et al., 1998). About 50% of all heart failure cases is found in patients older than 70 (Roger et al., 2011). Although nowadays an important decrease in the mortality for heart disease, cardiovascular disease is the most frequent single cause of death in the elderly population. The prognosis of heart failure among elderly is poor with a 4 year survival of only around 50% (Cacciatore et al., 1998) heart failure is also one of the most important causes of comorbidity and hospitalization rising health costs.

It is important to underline that heart failure in the elderly appears when cardiovascular structural and functional age-related changes are already evident. Moreover, the age-associated changes in cardiovascular structure/function are involved in the increased risk for heart failure in older people. An age-dependent increase in left ventricular wall thickness in men and woman without hypertension has been described in the Framingham Heart Study and Baltimore Longitudinal Study on Aging (Ho et al., 1993). These modifications in structure of heart are characterized by an increase in cardiomyocyte size and myocardial thickness modifying the contractile efficiency and increasing left ventricular mass (Olivetti et al., 1991; Khouri et al., 2005). As a result of age-related chronic stress, the myocardium can undergo cardiomyocyte death including necrosis, apoptosis or autophagy. This process induces initially a compensatory remodeling characterized by the alterations of extracellular matrix (ECM) composition involving the synthesis of myofibroblasts, the degradation of collagen through TGF-B signaling. These alterations lead to the hypertrophy of the remaining cells and to pathologic remodeling, with consequent reactive fibrosis that increases cardiac stiffness and reduces the cardiac compliance (Boyle et al., 2011).

It has been reported that the process of cardiac aging, as well as the progressive heart failure, is also characterized by the impaired Ca^++^ reuptake and the decreased SR Ca^++^ storage. In some studies this decline has been explained by the modification of SERCA_2_ protein. This impaired Ca^++^ reuptake is responsible for the delays in the ventricular relaxation (Sucharov et al., 2006). The myocardial remodeling, myocardial stiffening and the delayed ventricular relaxation compromise the left ventricular filling in early diastole. In order to maintain an adequate left ventricular filling in elderly the atrial contraction (A wave) is further increased leading to the atrial hypertrophy increasing so the risk for atrial fibrillation. However, these data have not been confirmed utilizing other epidemiological and autopsy-based studies (**?**) in subjects free from coronary artery disease and hypertension. In fact, these studies have demonstrated no significant changes in cardiac mass in women and a decrease in left ventricular mass in men. Based on these and other studies it has been shown that there is no significant changes in left ventricular mass in the elderly women, but there is a reduction of cardiac mass in men (cardiac sarcopenia). Regarding to this Lin et al. (2008) demonstrated, combining experimental and mathematical models, in mice an age-related cardiac sarcopenia and that LV remodeling due to increased end diastolic pressure could be an underlying mechanism for age-related LV dysfunction. Senescence is characterized by a reduction of myocytes cell number. In order to maintain the cardiac function, some compensatory mechanisms are induced such as: increase of individual myoctye size, increase of intracellular glycogen storage and reactive fibrosis. This changes induce a depressed cardiac function in elderly. Moreover, asymmetric increase in the intraventricular septum does not influence the total cardiac mass. Nevertheless these changes do not modify significantly the systolic cardiac function at rest as demonstrated by echocardiographic and radionuclide studies in normotensive healthy population (Hess et al., 2002; Khouri et al., 2005). Instead the pattern of diastolic function changes with aging and plays a pivotal role in increased prevalence of diastolic heart failure in the elderly. There are many similarities between the aging and failing heart. For example, morphologically myocytes hypertrophy and cardiac fibrosis occur in both aging and failing heart that exhibit decreased diastolic function and increased ventricular mass. In both there is a decreased functional reserve and defective cardiac energetics. The age-related changes in β-AR behavior are quite similar to those related to failing heart and these changes may be induced by the compensatory adrenergic drive activation. The stimulation of adrenergic receptors by catecholamines is the most important regulatory mechanism for cardiovascular performance. It is well known that the levels of cathecolamines are directly correlated with mortality and functional class of heart failure (Cohn et al., 1984). The cardiac β-receptors, in particular the β_1_ subtype, are downregulated in heart failure and the heart failure-associated reduced cardiac adrenergic responsiveness are related to an increase in G_α*i*_ activity and in activity of the adrenoreceptor kinases. Failing heart-associated β-AR down-regulation seems to effect the β_1_-AR but not the β_2_-AR density and this abnormality is also related to GRK_2_ and G_*i*_ up-regulation (Bristow et al., 1986). Physical performance as well as other stressors stimulate the adrenergic system increasing heart rate, myocardial contractility and relaxation, reducing left ventricular afterload and redistributing blood to working muscle. As above described, this pattern modifies during aging. A possible explanation of this behavior is in relationship with the chronic and gradual sympathetic hyperactivity. The association of failing to aging heart physiologic changes is not important only for the worldwide health care system but also for explaining the worst of quality of life in these patients. For these reasons since about 30 years the research studies about the relationship between adrenergic system and heart failure are interestingly increasing. In the early 1982, Bristow et al. (1982) studying the myocardium contractility “*in vivo*,” examined the β-AR pathway in failing hearts obtained from human subjects undergoing cardiac-transplantation. It was noticed an important reduction in the contractile response to isoproterenol in these “*in vivo*” cardiac cells and also an evident reduction of the β-AR density. The same author (Bristow et al., 1986) after a couple of years, developed a radiolig and biopsy method for identifying β-AR in human ventricular myocardium. This method it is thought to be useful in the direct analyses in the β-AR density and in the study of down-regulation. The development of this new method opened a new window in the molecular pharmacology of adrenergic system. Brodde et al. (1989) concluded that the β-AR density level reduction is associated to the severity of congestive heart failure, and in valvular heart failure exist a decrease in either β_1_-AR and β_2_-AR density. β-AR desensitization in the failing heart is proved from many authors, but this is not the one and only mechanism involved in a complex disease like heart failure. Feldman et al. (1988) observed the increased activity of α G40 complex in the human failing heart and considered as a possible new marker for the severity of failing heart. Fu et al. (1992) also concluded that the role of G_*i*_ protein is crucial in the failing heart but it was observed an increase in functional activity rather than in its amount. In 1997 Ping et al. (1997) during the pacing induced congestive heart failure (CHF) examined the alteration of β-ARs, AC activity and GRKs. They concluded that in the advanced stages of CHF, β-ARs and AC are down-regulated while there is an increase in the GRKs levels since the early stages of CHF. Ungerer et al. (1993) studying the involvement of β-AR in human heart failure models found for the first time an inverse correlation between β-AR density and β-ARKs. The β-AR density is reduced but also the remaining receptors are less effective. Initially the catecholamines released from sympathetic nerves try to restore the cardiac functioning by acting on the β-AR. In long term the enhanced sympathetic drive causes the β-AR desensitization, At least part of this adaptative mechanism should protect heart from further sympathetic activation, but the overall cardiac performance is depressed.

The genetic engineering and the need to establish a quite similar model of human disease have driven the scientific research to develop transgenic animal models. These model are produced by using DNA microinjections in animals or by using another methodology like the gene knock-out models. In 1996 Rockman et al. (1996) in order to investigate the β-AR alteration and contribution in heart failure, used a model of transgenic mice with overexpression of β-ARK_1_ inhibitors or β_2_-AR overexpression. It resulted that overexpressed β-ARK_1_ inhibitors increased the myocardial contractility and prevented the development of heart failure. A couple of years later Harding et al. (2001) developed a transgenic mice model overexpressing β-ARK_1_ inhibitor (β-ARK_1CT_) and calsequestrin protein. In this model a treatment with β-blockers led to an improvement of cardiac contractile functioning and an longer survival.

The development of animal surgery techniques also helped to obtain similar rat models of heart failure. Vinge et al. (2001) studied the involvement of GRKs in postinfarction heart failure model in rats undergoing ligation of left coronary artery. They concluded that GRK_2_ and β-arrestin_1_ are primary regulators in the endothelial function in the heart failure, while the GRK_3_ and GRK_5_ play a very important role in the cardiac myocyte functioning. The increased levels of GRK_2_was related to the infarction induction suggesting that this protein may precipitate the development of acute heart failure. Iaccarino et al. (2005) found an inverse correlation between the β-ARs expression and the levels of GRK_2_. This inverse correlation can be explained by the phsophorylation of β-ARs by GRKs and furthermore their internalization and desensitization process. Interestingly the level of GRK_2_ is associated to the degree of heart failure, suggesting an importance of GRKs as a heart failure progression biomarker. In 2007, for the first time, Lymperopoulos et al. (2007) studied the involvement of adrenal signaling in the pathophysiology of heart failure. The study was performed in two different models, in transgenic mice with cardiac overexpression of SR calcium binding protein calsequestrin (CSQ) and rat model undergoing myocardial infarction. It was noticed an α_2_-AR down-regulation independent of species and a GRK_2_ up-regulation. In 2012, in a post-infarction heart failure rat model Rengo et al. (2012b) studied the influence of adrenergic blockade in the in GRK_2_ and α_2_-AR adrenal medulla dysregulation. It has been confirmed that there is an up-regulation of adrenal medulla GRK_2_ and a down-regulation of α_2_-AR and that their blockade with β-blockers normalizes the level of GRK_2_. The same author (Rengo et al., 2012c) in 2012 investigated the role of β_2_-AR in a post-myocardial infarction heart failure rat model. This model underwent an adenoviral-mediated overexpression of β_2_-AR after 4 weeks of surgery procedure. It was observed that the overexpression of β_2_-AR improves the angiogenesis process and enhanced the coronary reserve and myocardial blood flow. This pro-angiogenic characteristic of β_2_-AR was associated to the activation of pro-angiogenic vascular endothelial growth factor, protein kinase B, endothelial nitric oxide synthase VEGF/PKB/eNOS pathway.

Recently returning to the well-known method of detection GRKs levels from the lymphocytes, Rengo et al. (2014) performed a prospective study in a group of heart failure patients who underwent exercise training. It was shown for the first time that the subjects after physical performance presented reduced levels of GRKs and also predicted survival. In Table 2 the main general failing heart-related changes in β-adrenergic signaling are described.

**Table 2:** **Failing heart: the main general changes in β-adrenergic signaling**.

**Author**	**Constituent**	**Species**	**Changes**
Bristow et al., 1982	β-AR density	Human (heart)	Decreased
Bristow et al., 1986	β_1_-AR density	Human (heart)	Decreased
Brodde et al., 1989	β_1, 2_-AR density	Human (heart	Decreased
Feldman et al., 1988	α G_40_ activity	Human (heart)	Increased
Ungerer et al., 1993	β-ARK	Human (heart)	Up-regulated
Fu et al., 1992	G_*i*_	Human (heart)	Increased
Ping et al., 1997	β-AR	Human (heart)	Down-regulated
Ping et al., 1997	AC	Human (heart)	Down-regulated
Ping et al., 1997	GRKs	Human (heart)	Increased
Rockman et al., 1996	β-ARK_1_ Inhibitors (overexpression)	Transgenic mouse (heart)	Increased contractility
Harding et al., 2001	β-ARKct (overexpression)	Transgenic mouse (heart)	Increased contractility
Vinge et al., 2001	β-ARRESTIN_1_	Rat (heart)	Increased
Vinge et al., 2001	GRK_2_	Rat (heart)	Increased
Vinge et al., 2001	GRK_5_	Rat (heart)	Increased
Iaccarino et al., 2005	GRK2	Human (heart)	Up-regulated
Lymperopoulos et al., 2007	α_2_-AR	Rat (adrenal medulla)	Down-regulated
Lymperopoulos et al., 2007	α_2_-AR	Transgenic mouse (adrenal medulla)	Down-regulated
Lymperopoulos et al., 2007	GRK_2_	Rat (adrenal medulla)	Up-regulated
Lymperopoulos et al., 2007	GRK_2_	Transgenic mouse (adrenal medulla)	Up-regulated
Rengo et al., 2012a,b,c	α_2_-AR density	Rat (adrenal medulla)	Down-regulated
Rengo et al., 2012a,b,c	GRK_2_	Rat (adrenal medulla)	Increased
Rengo et al., 2014	GRK_2_	Human (lymphocytes)	Increase

## Conclusions

Cardiovascular diseases are the most common cause of death in elderly population. Increased longevity is associated to an increased heart failure morbidity with poor prognosis among elderly. For this reasons it is very important to understand and clarify the pathophysiological mechanisms that underlay the aging heart process. From the present literature it seems that “β-adrenoceptor desensitization/down-regulation” is a general and common mechanism which explains age- and heart failure-related decrease in β-adrenoceptor response to agonists. In particular, in both aging and failing hearts the decrease in β-adrenoceptor responsiveness is related to changes in G-proteins and kinases activity, although there are differences in the level and the extent of these changes among different studied species. These molecular alterations are responsible for the most structural and functional changes in aging heart. The improved understanding of this mechanism could help in the future to develop new therapy approach and ameliorate life quality among elderly population.

### Conflict of interest statement

The authors declare that the research was conducted in the absence of any commercial or financial relationships that could be construed as a potential conflict of interest.

## References

[B1] Abrass I. B. Scapace P. J. (1981). Human lymphocytes β-adrenergic receptors are unaltered with age. J. Gerontol. 36, 298–301 10.1093/geronj/36.3.298 6262399

[B2] Alquist R. F. (1948). A study of the adrenotropic receptors. Am. J. Physiol. 153, 586–600 18882199 10.1152/ajplegacy.1948.153.3.586

[B3] Altschuld R. A. Starling R. C. Hamlin R. L. Billman G. E. Hensley J. Castillo L. (1995). Response of failing canine and human heart cells to β_2_-adrenergic stimulation. Circulation 92, 1612–1618 10.1161/01.CIR.92.6.1612 7664448

[B4] Bazan A. Van de Velde E. Fraeyman N. (1994). Effects of age on β-receptors. G_*s*__α_ - and G_*i*α_ - proteins in rat heart. Res. Pharmacol. 48, 479–486 10.1016/0006-2952(94)90277-1 8068035

[B5] Benovic J. L. Onorato J. J. Arriza J. L. Stone W. C. Lohse M. Jenkins N. A. (1991). Cloning, expression and chromosomal localization of β-adrenergic receptor kinase 2. J. Biol. Chem. 266, 14939–14946 1869533

[B6] Bogoyevitch M. A. Andersson M. B. Gillespie-Brown J. Clerck A. Glennon P. E. Fuller S. J. (1996). Adrenergic receptor stimulation of the mitogen-activated protein kinase cascade and cardiac hypertrophy. Biochem. J. 314, 115–121 8660271 10.1042/bj3140115 PMC1217013

[B7] Bohm M. Dorner H. Htun P. Lensche H. Platt D. Erdmann E. (1993). Effects of exercise on myocardial adenylatecyclase and G_*i*_ alpha expression in senescence. Am. J. Physiol. 264, H805–H814 8384423 10.1152/ajpheart.1993.264.3.H805

[B8] Borea P. A. Amerini S. Masini I. Cerbai E. Ledda F. Mantelli L. (1992). β_1_ and β_2−_ adrenoceptors in sheep cardiac ventricular muscle. J. Mol. Cell. Cardiol. 24, 753–763 10.1016/0022-2828(92)93389-2 1357182

[B9] Boyle A. J. Shih H. Hwang J. Ye J. Lee B. Zhang Y. (2011). Cardiomyopathy of aging in the mammalian heart is characterized by myocardial hypertrophy, fibrosis and a predisposition towards cardiomyocyte apoptosis and autophagy. Exp. Gerontol. 46, 549–559 10.1016/j.exger.2011.02.010 21377520 PMC3104129

[B10] Bristow M. R. Ginsburg R. Minobe W. Cubicciotti R. S. Sageman W. S. Lurie K. (1982). Decreased catecholamine sensitivity and β-adrenergic-receptor density in failing human hearts. N. Engl. J. Med. 307, 205–211 10.1056/NEJM198207223070401 6283349

[B11] Bristow M. R. Ginsburg R. Umans V. Fowler M. Minobe W. Rasmussen R. (1986). β_1_ and β_2_-adrenergic-receptor subpopulations in non failing and failing human ventricular myocardium: coupling of both receptorsubtypes to muscle contraction and selective β_1_-receptor downregulation in heart failure. Circ. Res. 59, 297–309 10.1161/01.RES.59.3.297 2876788

[B12] Brodde O. E. Zerkowski H. R. Doetsch N. Motomura S. Khamssi M. Michel M. C. (1989). Myocardial β-adrenoceptor changes in heart failure: concomitant reduction in β_1_- and β_2_-adrenoceptor function related to the degree of heart failure in patients with mitral valve disease. J. Am. Coll. Cardiol. 14, 323–331 10.1016/0735-1097(89)90181-2 2569001

[B13] Buxton L. O. Bruton L. L. (1985). Direct analyses of β-adrenergic receptor subtype in intact adult ventricular myocytes of the rats. Circ. Res. 56, 126–132 10.1161/01.RES.56.1.126 2857116

[B14] Cacciatore F. Gallo C. Ferrara N. Abete P. Paolisso G. Canonico S. (1998). Morbidity patterns in aged population in southern Italy. A survey sampling. Arch. Gerontol. Geriatr. 26, 201–213 10.1016/S0167-4943(98)00003-X 18653137

[B15] Cannavo A. Rengo G. Liccardo D. Pironti G. Scimia M. C. Scudiero L. (2013). Prothymosin alpha protects cardiomyocytes againts ischemia-induced apoptosis via presentation of Act activation. Apoptosis. 18, 1252–1261 10.1007/s10495-013-0876-9 23857453

[B16] Cerbai E. Varani K. Barbieri M. Borea P. A. Mugelli A. (1995). β-adrenoreceptor subtypes in young and old rat ventricular myocytes. A combined patch-clamp and binding study. Br. J. Pharmacol. 116, 1835–1842 10.1111/j.1476-5381.1995.tb16671.x 8528568 PMC1909098

[B17] Cohn J. N. Levine T. B. Olivari M. T. Garberg V. Lura D. Francis G. S. (1984). Plasma norepinephrine as a guide to prognosis in patients with chronic congestive heart failure. N. Engl. J. Med. 311, 819–823 10.1056/NEJM198409273111303 6382011

[B18] Corbi G. Acanfora D. Iannuzzi G. L. Longobardi G. Cacciatore F. Furgi G. (2008). Hypermagnesemia predicts mortality in elderly with congestive heart disease: relationship with laxative and antacid use. Rejuvenat. Res. 11, 129–138 10.1089/rej.2007.0583 18279030

[B19] Corbi G. Conti V. Russomanno G. Longobardi G. Furgi G. Filippelli A. (2013a). Adrenergic signaling and oxidative stress: a role of sirtuins? Front. Physiol. 4:324 10.3389/fphys.2013.00324 24265619 PMC3820966

[B20] Corbi G. Bianoc A. Turchiarelli V. Cellurale M. Fatica F. Daniele A. (2013b). Potential mechanisms linking atherosclerosis and increased cardiovascular risk in COPD: focus on sirtuins. Int. J. Mol. Sci. 14, 12696–12713 10.3390/ijms140612696 23774840 PMC3709808

[B21] Corbi G. Conti V. Russomanno G. Rengo G. Vitulli P. Ciccarelli A. L. (2012a). Is physical activity able to modify oxidative damage in cardiovascular aging? Oxid. Med. Cell. Longev. 2012, 728547 10.1155/2012/728547 23029599 PMC3458405

[B22] Corbi G. Conti V. Scapagnini G. Filippelli A. Ferrara N. (2012b). Role of sirtuins, calorie restriction and physical activity in aging. Front. Biosci. (Elite. Ed). 4:417 10.2741/417 22201912

[B23] Corbi G. M. Carbone S. Ziccardi P. Giugliano G. Marfella R. Nappo F. (2002). FFAs and QT intervals in obese women with visceral adiposity: effects of sustained weight loss over 1 year. J. Clin. Endocrinol. Metab. 87, 2080–2083 10.1210/jc.87.5.2080 11994344

[B24] Davies C. H. Ferrara N. Harding S. E. (1996). β-Adrenoceptor function changes with age of subject in myocytes from non-failing human ventricle. Cardiovasc. Res. 31, 152–156 10.1016/0008-6363(95)00187-5 8849600

[B25] Dobson J. G. Fenton R. A. Romano F. D. (1990). Increased myocardial adenosine production and reduction of β-adrenergic contractile response in aged hearts. Cir. Res. 66, 1381–1390 10.1161/01.RES.66.5.1381 2159390

[B26] Ehsani A. A. (1987). Cardiovascular adaptations to exercise training in the elderly. Fed. Proc. 46, 1840–1843 3556605

[B27] Enns L. C. Morton J. F. Treuting P. R. Edmond M. J. Wolf N. S. Dai D. F. (2009). Disruption of protein kinase A in mice enhances healthy aging. PLoS ONE 4:e5963 10.1371/journal.pone.0005963 19536287 PMC2693670

[B28] Esler M. D. Turner A. G. Kaye A. G. Thompson J. M. Kingwell B. A. Morris M. (1995). Aging effects on human sympathetic neuronal function. Am. J. Physiol. 268(1 Pt), R278–R285 7840332 10.1152/ajpregu.1995.268.1.R278

[B29] Feldman A. M. Cates A. E. Veazey W. B. Hershberger R. E. Bristow M. R. Baughman K. L. (1988). Increase of the 40,000-mol wt pertussis toxin substrate (G protein) in the failing human heart. J. Clin. Invest. 82, 189–197 10.1172/JCI113569 2839545 PMC303493

[B30] Ferrara N. Abete P. Corbi G. Paolisso G. Longobardi G. Calabrese C. (2005). Insulin-induced changes in β-adrenergic response: an experimental study in the isolated rat papillary muscle. Am. J. Hypertens. 18, 348–353 10.1016/j.amjhyper.2004.10.006 15797652

[B31] Ferrara N. Bohm M. Zolk O. O'Gara P. Harding S. E. (1997a). The role of G_*i*_-proteins and β-adrenoceptors in the age-related decline of contraction in guinea-pig ventricular myocytes. J. Mol. Cell. Cardiol. 29, 439–448 10.1006/jmcc.1996.0397 9140804

[B32] Ferrara N. Davia K. Abete P. Rengo F. Harding S. E. (1997b). Alterations in β-adrenoceptor mechanisms in the aging heart. Relationship with heart failure. Aging (Milano). 9, 391–403 9553617 10.1007/BF03339620

[B33] Ferrara N. Corbi G. Bosimini E. Cobelli F. Furgi G. Giannuzzi P. (2006). Cardiac rehabilitation in the elderly: patient selection and outcomes. Am. J. Geriatr. Cardiol. 15, 22–27 10.1111/j.1076-7460.2006.05289.x 16415643

[B34] Ferrara N. O'Gara P. Wynne D. G. Brown L. A. del Monte F. Poole-Wilson P. A. (1995). Decreased contractile responses to isoproterenol in isolated cardiac myocytes from aging guinea-pigs. J. Mol. Cell. Cardiol. 27, 1141–1150 10.1016/0022-2828(95)90050-0 7473772

[B35] Fleg J. L. Schulman S. O'Connor F. Becker L. C. Gerstenblith G. Clulow J. F. (1994). Effects of acute β-adrenergic receptor blockade on age associated changes in cardiovascular performance during dynamic exercise. Circulation 90, 2333–2341 10.1161/01.CIR.90.5.2333 7955191

[B36] Folkow B. Svanborg A. (1993). Physiology of cardiovascular aging. Physiol. Rev. 73, 725–764 8105498 10.1152/physrev.1993.73.4.725

[B37] Freedman N. J. Liggett S. B. Drachman D. E. Pei G. Caron M. G. Lefkowtiz R. J. (1995). Phosphorylation and desensitization of the human β_1_-adrenergic receptor: involvement of G protein coupled receptor kinase and cAMP dependent protein kinase. J. Biol. Chem. 270, 17953–17961 7629102 10.1074/jbc.270.30.17953

[B38] Froehlich J. P. Lakatta E. G. Beard E. Spurgeon H. A. Weisfeldt M. L. Gerstenbilth G. (1978). Studies of sarcoplasmic reticulum function and contraction duration in young and aged rat myocardium. J. Mol. Cell. Cardiol. 10, 427–438 10.1016/0022-2828(78)90364-4 660657

[B39] Fu L. X. Liang Q. M. Waagstein F. Hoebeke J. Sylvén C. Jansson E. (1992). Increase in functional activity rather than in amount of G_*i*_-alpha in failing human heart with dilated cardiomyopathy. Cardiovasc. Res. 26, 950–955 10.1093/cvr/26.10.950 1336712

[B40] Gudmundsdottir E. Benediktsdottir V. E. Gudbjarnason S. (1991). Combined effects of age and dietary fat on β-1 receptors and Ca2+ channels in rat hearts. Am. J. Physiol. 260(1 Pt 2), H66–H72 1847019 10.1152/ajpheart.1991.260.1.H66

[B41] Hall R. A. Premont R. T. Chow C. W. Blitzer J. T. Pitcher J. A. Claing A. (1998). The β_2_-adrenergic receptor interacts with the Na^+/H^+^-exchanger^ regulatory factor to control Na^+^/H^+^ exchange. Nature 392, 626–630 10.1038/33458 9560162

[B42] Harding V. B. Jones L. R. Lefkowitz R. J. Koch W. J. Rockman H. A. (2001). Cardiac βARK_1_ inhibition prolongs survival and augments β-blocker therapy in a mouse model of severe heart failure. Proc. Natl. Acad. Sci. U.S.A. 98, 5809–5814 10.1073/pnas.091102398 11331748 PMC33295

[B43] Hess P. S. Fleg J. Lakatta E. G. Shapiro E. P. (2002). Left ventricular remodeling with age in normal men versus women: novel insights using three-dimensional magnetic resonance imaging. Am. J. Cardiol. 90, 1231–1236 10.1016/S0002-9149(02)02840-0 12450604

[B44] Ho K. K. Pinsky J. L. Kannel W. B. Levy D. (1993). The epidemiology of heart failure: the Framingham study. J. Am. Coll. Cardiol. 22(4 Suppl. A), 6A–13A 10.1016/0735-1097(93)90455-A 8376698

[B45] Iaccarino G. Barbato E. Cipolletta E. De Amicis V. Margulies K. B. Leosco D. (2005). Elevated myocardial and lymphocyte GRK2 expression and activity in human heart failure. Eur. Heart J. 17, 1752–1758 10.1093/eurheartj/ehi429 16055494

[B46] Jiang M. T. Moffat M. P. Narayanan N. (1993). Age-related alterations in the phosphorylation of sarcoplasmic reticulum and myofibrillar proteins and diminished contractile response to isoproterenol in intact rat ventricle. Circ. Res. 72, 102–111 10.1161/01.RES.72.1.102 8380258

[B47] Khouri M. G. Maurer M. S. El-Khoury Rumbarger L. (2005). Assessment of age-related changes in left ventricular structure by freehand three-dimensional echocardiography. Am. J. Geriatr. Cardiol. 14, 118–125 10.1111/j.1076-7460.2005.03845.x 15886537

[B48] Kuschel M. Zhou Y. Y. Spurgeon H. A. Bartel S. Karchewski P. Zhang S. J. (1999). β_2_-Adrenergic cAMP signaling is uncoupled from phosphorylation of cytoplasmic proteins in canine heart. Circulation. 99, 2458–2465 10.1161/01.CIR.99.18.2458 10318670

[B49] Lakatta E. G. Levy D. (2003). Arterial and cardiac aging: major shareholders in cardiovascular disease enterprises: part II: the aging heart in health: links to heart disease. Circulation 107, 346–354 10.1161/01.CIR.0000048893.62841.F7 12538439

[B50] Landmann R. Bittinger H. Buhler F. R. (1981). High affinity β_2_-adrenergic receptors in mononuclear leucocytes: similar density in young and old normal subjects. Life Sci. 29, 1761–1771 10.1016/0024-3205(81)90186-7 6272047

[B51] Leineweber K. Wangemann T. Giessler C. Bruck H. Dhein S. Kostelka M. (2002). Age-dependent changes of cardiac neuronal noradrenaline reuptake transporter (uptake1) in human heart. Am. Coll. Cardiol. 40, 1459–1465 10.1016/S0735-1097(02)02168-X 12392837

[B52] Leinweber K. Klapproth S. Beilfuss A. Silber R. E. Heusch G. Philipp T. (2003). Unchanged G-protein-coupled receptor kinase activity in the aging human heart. J. Am. Col. Cardiol. 42, 1487–1492 10.1016/S0735-1097(03)01063-5 14563597

[B53] Lemoine H. Kaumann A. J. (1991). Regional differences of β_1_- and β_2_-adrenoceptor-mediated functions in feline heart: a β-adrenoceptormediated positive inotropic effect possibly unrelated to cyclic AMP. Naunyn Schmiedebergs. Arch. Pharmacol. 344, 56–59 1685558 10.1007/BF00167383

[B53a] Leosco D. Iaccarino G. Cipolletta E. De Santis D. Pisani E. Trimarco V. (2003). Exercise restores β-adrenergic vasorelaxation in aged rat carotid arteries. Am. J. Physiol. Heart. Circ. Physiol. 285, 369–374 10.1152/ajpheart.00019.2003 12637361

[B54] Leosco D. Rengo G. Iaccarino G. Filippelli A. Lymperopoulos A. Zincarelli C. (2007). Exercise training and β-blocker treatment ameliorate age-dependent impairment of β-adrenergic receptor signaling and enhance cardiac responsiveness to adrenergic stimulation. Am. J. Physiol. Heart Circ. 293, H1596–H1603 10.1152/ajpheart.00308.2007 17557919

[B55] Lewis C. J. Haibin G. Brown M. J. Harding S. E. (2004). Overexpression of β_1_-adrenoceptors in adult rat ventricular myocytes enhances CGP 12177A cardiostimulation: implications for “putative” β_4_-adrenoceptor pharmacology. Br. J. Pharmacol. 141, 813–824 10.1038/sj.bjp.0705668 14757703 PMC1574257

[B56] Lin J. Lopez E. F. Jin Y. Van Remmen H. Bauch T. Han H. C. (2008). Age-related cardiac muscle sarcopenia: combining experimental and mathematical modeling to identify mechanisms. Exp. Gerontol. 43, 296–306 10.1016/j.exger.2007.12.005 18221848 PMC2323436

[B57] Lonardo G. Cerbai E. Casini S. Giunti G. Bonacchi M. Battaglia F. (2005). Pharmacological modulation of the hyperpolarization-activated current (I f) in human atrial myocytes: focus on G protein-coupled receptors. J. Mol. Cell. Cardiol. 38, 453–460 10.1016/j.yjmcc.2004.12.010 15733905

[B58] Lymperopoulos A. Rengo G. Funakoshi H. Eckhart A. D. Koch W. J. (2007). Adrenal GRK_2_ mediates sympathetic overdrive in heart failure. Nat. Med. 13, 315–323 10.1038/nm1553 17322894

[B59] Marshall C. J. (1995). Specificity of receptor tyrosine kinase signaling: transient versus sustained extracellular signal-regulated kinase activation. Cell 80, 179–185 10.1016/0092-8674(95)90401-8 7834738

[B60] McConnachie G. Langeberg L. K. Scott J. D. (2006). AKAP signaling complexes: getting to the heart of the matter. Trends Mol. Med. 12, 317–323 10.1016/j.molmed.2006.05.008 16809066

[B61] Morisco C. Zebrowski D. Condorelli G. Tsichlis P. Vatner S. F. Sadoshima J. (2000). The Akt-glycogen synthase kinase 3β pathway regulates transcription of atrial natriuretic factor induced by β-adrenergic receptor stimulation in cardiac myocytes. J. Biol. Chem. 275, 14466–14475 10.1074/jbc.275.19.14466 10799529

[B62] Ng A. V. Callister D. G. Johnson D. G. Seals D. R. (1993). Age and gender influence muscle sympathetic nerve activity at rest in healthy humans. Hypertension. 21, 498–503 10.1161/01.HYP.21.4.498 8458648

[B63] Olivetti G. Melissari M. Capasso J. M. Anversa P. (1991). Cadiomyopathy of aging human heart. Myocyte loss reactive cellular hypertrophy. Circ. Res. 68, 1560–1568 10.1161/01.RES.68.6.1560 2036710

[B64] Ping P. Anzai T. Gao M. Hammond H. K. (1997). Adenyl cyclase and G protein receptor kinase expression during development of heart failure. Am. J. Physiol. 273(2 Pt 2), H707–H717 9277487 10.1152/ajpheart.1997.273.2.H707

[B65] Ramirez M. T. Zhao X. L. Schulman H. Brown J. H. (1997). The nuclear δ B isoform of Ca^2+^/calmodulin-dependent protein kinase regulates atrial natriuretic factor gene expression in ventricular myocytes. J. Biol. Chem. 272, 31203–31208 10.1074/jbc.272.49.31203 9388275

[B66] Rengo G. Galasso G. Femminella G. D. Parisi V. Zincarelli C. Pagano G. (2014). Reduction of lymphocyte G protein-coupled receptor kinase-2 (GRK2) after exercise training predicts survival in patients with heart failure. Eur. J. Prev. Cardiol. 21, 4–11 10.1177/2047487313491656 23689525

[B67] Rengo G. Perrone-Filardi P. Femminella G. D. Liccardo D. Zincarelli C. de Lucia C. (2012a). Targeting the β-adrenergic receptor system through G-protein coupled receptor kinase 2: a new paradigm from therapy and prognostic evaluation in heart failure: from bench to bedside. Circ. Heart Fail. 5, 358–391 10.1161/CIRCHEARTFAILURE.112.966895 22589366

[B68] Rengo G. Lymperopoulos A. Zincarelli C. Femminella G. Liccardo D. Pagano G. (2012b). Blockade of β-adrenoceptors restores the GRK_2_-mediated adrenal α2-adrenoceptor-catecholamine production axis in heart failure. Br. J. Pharmacol. 166, 2430–2440 10.1111/j.1476-5381.2012.01972.x 22519418 PMC3448904

[B69] Rengo G. Zincarelli C. Femminella G. D. Liccardo D. Pagano G. de Lucia C. (2012c). Myocardial β_2_-adrenoceptor gene delivery promotes coordinated cardiac adaptive remodelling and angiogenesis in heart failure. Br. J. Pharmacol. 166, 2348–2361 10.1111/j.1476-5381.2012.01954.x 22452704 PMC3448898

[B70] Rinaldi B. Corbi G. Boccuti S. Filippelli W. Rengo G. Leosco D. (2006). Exercise training affects age-induced changes in SOD and heat shock protein expression in rat heart. Exp. Gerontol. 41, 764–770 10.1016/j.exger.2006.05.008 16822632

[B71] Rockman H. A. Koch W. J. Lefkowitz R. J. (2002). Seven-transmembrane-spanning receptors and heart function. Nature 415, 206–212 10.1038/415206a 11805844

[B72] Rockman H. A. Koch W. J. Milano C. A. Lefkowitz R. J. (1996). Myocardial β-adrenergic receptor signaling “*in vivo*”: insights from transgenic mice. J. Mol. Med. (Berl.) 74, 489–495 10.1007/BF00204974 8892053

[B73] Roger V. L. Go A. S. Lloyd-Jones D. M. Benjamin E. J. Berry J. D. Borden W. B. (2011). Executive summary: heart disease and stroke statistics—2011 update: a report from the american heart association. Circulation 123, 459–463 10.1161/CIR.0b013e3182009701 22215894

[B74] Scarpace P. J. Shu Y. Tumer N. (1994). Influence of exercise training on myocardial-adrenergic signal transduction: differential regulation with age. J. Appl. Physiol. 77, 737–741 8002522 10.1152/jappl.1994.77.2.737

[B75] Scholtz D. G. Kitzman D. W. Hagen P. T. Ilstrup D. M. Edwards W. D. (1988). Age-related changes in normal human hearts during the first 10 decades of life. Part I (Growth): a quantitative anatomic study of 200 specimens from subjects from birth to 19 years old. Mayo Clin. Proc. 63, 126–136 10.1016/S0025-6196(12)64945-3 3276973

[B77] Schutzer W. E. Reed J. F. Bliziotes M. Mader S. L. (2001). Up-regulation of G protein-linked receptor kinases with advancing age in rat aorta. Am. J. Physiol. Regul. Integr. Comp. Physiol. 280, R897–R903 11171671 10.1152/ajpregu.2001.280.3.R897

[B78] Shioi T. Inuzuka Y. (2012). Aging as a substrate of heart failure. J. Cardiol. 60, 423–428 10.1016/j.jjcc.2012.07.015 23068289

[B78a] Spindler M. J. Burmeister B. T. Huang Y. Hsiao E. C. Salomonis N. Scott M. J. (2013). AKAP13 Rho-GEF and PKD-binding domain deficient mice develop normally but have an abnormal response to β-adrenergic-induced cardiac hypertrophy. PLoS ONE 8:e62705 10.1371/journal.pone.0062705 23658642 PMC3637253

[B79] Sucharov C. C. Mariner P. D. Nunley K. R. Long C. Leinwand L. Bristow M. R. (2006). A β_1_-adrenergic receptor CaM kinase II-dependent pathway mediates cardiac myocyte fetal gene induction. Am. J. Physiol. Heart Circ. Physiol. 291, H1299–H1308 10.1152/ajpheart.00017.2006 16501029

[B80] Ungerer M. Bohm M. Elce J. S. Erdmann E. Lohse M. J. (1993). Altered expression of β-adrenergic receptor kinase and β_1_-adrenergic receptors in failing human heart. Circulation 87, 454–463 10.1161/01.CIR.87.2.454 8381058

[B81] Van Biesen T. Hawes B. E. Luttrell D. K. Krueger K. M. Touhara K. Porfiri E. (1995). Receptor-tyrosine-kinase- and G β gamma-mediated MAP kinase activation by a common signaling pathway. Nature. 376, 781–784 10.1038/376781a0 7651538

[B82] Vinge L. E. Øie E. Andersson Y. Grøgaard H. K. Andersen G. Attramadal H. (2001). Myocardial dystribution and regulation of GRK and arrestin isoforms in congestive heart failure in rats. Am. J. Physiol. 281, H2490–H2499 11709416 10.1152/ajpheart.2001.281.6.H2490

[B82a] Wang J. Yang H. Hu X. Fu W. Xie J. Zhou X. (2013). Dobutamine-mediated heme oxygenase-1 induction via PI3K and p38 MAPK inhibits high mobility group box 1 protein release and attenuates rat myocardial ischemia/reperfusion injury *in vivo*. J. Surg. Res. 183, 509–516 10.1016/j.jss.2013.02.051 23531454

[B83] White M. Roden R. Minobe W. Khan M. F. Larrabee P. Wollmering M. (1994). Age-related changes in β-adrenergic neuroeffectors system in the human heart. Circulation 90, 1225–1238 10.1161/01.CIR.90.3.1225 8087932

[B84] Xiao R. P. Cheng H. Zhou Y. Y. Kuschel M. Lakatta E. G. (1999). Recent Advances in Cardiac β_2_-Adrenergic Signal Transduction Circ. Res. 85, 1092–1100 10.1161/01.RES.85.11.1092 10571541

[B85] Xiao R. P. Holh C. Altschuld R. Jones L. Livingston B. Ziman B. (1994). β_2_-adrenergic receptor- stimulated increase in cAMP in rat heart cells is not coupled to change Ca^2+^ dynamics, contractility or phospholamban phosphorylation. J. Biol. Chem. 269, 19151–19156 8034672

[B86] Xiao R. P. Ji X. Lakatta E. G. (1995). Functional coupling of the β_2_-adrenoceptor to a pertussis toxin-sensitive G protein in cardiac myocytes. Mol. Pharmacol. 47, 322–329 7870040

[B87] Xiao R. P. Tomhave E. D. Wang D. J. Ji X. Boluyt M. O. Cheng H. (1998). Age-associated reductions in cardiac β_1_- and β_2_-adrenergic responses without changes in inhibitory G proteins or receptor kinases. J. Clin. Invest. 101, 1273–1282 10.1172/JCI1335 9502768 PMC508681

[B88] Young J. B. Landsberg L. (1998). Ch. 17. Catecholamines and the adrenal medulla in Williams Textbook of Endocrinology, 9th Edn., eds Wilson J. D. Foster D. W. Kronenberg H. M. Larsen P. R. (Philadelphia, PA: W.B. Saunders), 665–728

[B89] Zhu W. Z. Zheng M. Koch W. J. Lefkowitz R. J. Kobilka B. K. Xiao R. P. (2001). Dual modulation of cell survival and cell death by β_2_-adrenergic signaling in adult mouse cardiac myocytes. Proc. Natl. Acad. Sci. U.S.A. 98, 1607–1612 10.1073/pnas.98.4.1607 11171998 PMC29304

